# The Depreciation of Medicine—Its Causes and Remedies

**Published:** 1906-09-22

**Authors:** 


### The Depreciation of Medicine—Its Causes and Remedies.

It is not at all uncommon among certain sections of the medical profession to hear expressions of surprise and regret on account of the slenderness of the influence which that profession, as a whole, exerts over the course of public affairs, or on account of what may perhaps be described, without exaggeration, as its political impotence. It follows from this impotence that no political party has ever thought it desirable to cultivate friendly relations with medical practitioners; and that the profession, as a whole, and even as represented by its most accomplished and personally most influential members, has never been able to obtain legislative sanction for reforms or enactments which every practitioner would regard as desirable. Departments of Government which are concerned with the protection of public health are still left in the hands of incapable or dishonest local authorities; a large proportion of the medical officers of health throughout the kingdom are not only powerless to enforce their recommendations, but even hold their offices at the pleasure of the proprietors of the nuisances which it is their business to condemn; and the wealthiest country in the world derives an ignoble revenue from casting the ægis of its protection over the often-times poisonous rubbish which is advertised and sold by the vilest and most dishonest of false pretenders to knowledge.

Stated baldly, and plainly, and in a single word, the cause of the evils thus indicated is the profound ignorance of the public upon all medical questions; and for this ignorance the medical profession is itself much to blame. The ignorance is largely promoted by the circumstance that there are considerable bodies of people—whole trades, indeed—who are actively interested in its maintenance, and who, like the silversmiths of Ephesus, have their living by making shrines for the idols of its worship. There is therefore a continual and active propagation of falsehood through most of the channels which are assumed to be open for the instruction of the community. Preachers attribute epidemics to supernatural causes; quack-medicine vendors ascribe personal maladies to harmless by-products of the animal economy, or pour forth pages of laudation of their combinations of alcohol with narcotics; and the editors of "religious" or other inferior newspapers—sometimes venal, sometimes only ignorant—are always ready to give insertion to lying paragraphs intended to undermine or to belittle medical authority. In the face of this quite systematic depreciation of medicine, the profession, hitherto, has done little or nothing in the way of self-defence. We do not want a few members of Parliament, pledged to support measures acceptable to small fractions of their constituents, but we want such a diffusion of knowledge as would serve to modify public opinion, to relegate quacks and impostors to their natural position of insignificance, and to bring medical knowledge into demand as the proper and only safe guide to the community upon medical questions. The profession should abandon its past attitude of contemptuous neglect of the enemies against whom it has to fight and assume the part of a public instructor. It is time for it to begin the task of "educating its masters"; and to do this without either being appalled at the extent of the leeway which it has to make up, or at the clamour by which its first efforts would be encountered by its enemies. In this country, as a rule, the medical profession seems to be either afraid or ashamed to assert itself; and, to take but a single example, an association professedly intended to promote physical culture as a means towards the preservation of health is content to accept a doubtless very well meaning bishop as its president and chief spokesman, and to receive from him what is little more than a dilution of facts and principles which the surgeons and physicians standing near him on the platform could have asserted at first hand from their personal knowledge and experience. The medical profession possesses an abundance of men who are both good speakers and masters of the subjects on which the public stand in need of instruction. It would be quite possible for such men in every considerable town in England to organise lectures and discussions which, in the course of a few years, would elevate the now neglected or contemned medical knowledge to its proper place as a guide both to Imperial legislation and to the administration of the law in the hundreds of localities in which it is at present suffered to remain dormant.

